# Spatiotemporal fluorescence imaging of microRNA activity in 3-D models of human epidermis reveals contribution of the Notch pathway in the regulation of miR-30a in aging skin

**DOI:** 10.1016/j.xjidi.2025.100444

**Published:** 2025-12-17

**Authors:** Alejandro Gonzalez Torres, Fabien P. Chevalier, Ruth Aquino, Mélanie Aimard, Patrick Baril, Jérôme Lamartine

**Affiliations:** 1 Laboratory of Tissue Biology and Therapeutic Engineering (LBTI), CNRS UMR5305 – University Claude Bernard Lyon I, Lyon, France; 2 Centre de Biophysique Moléculaire (CBM), CNRS UPR 4301 – University of Orléans, UFR Sciences & Techniques, Orleans, France

**Keywords:** Aging, Epidermis, microRNAs, Notch, RILES

## Abstract

MicroRNAs are short noncoding RNAs that play important roles in fine tuning genetic networks as genes post-transcriptional regulators. Monitoring the regulatory activity of microRNAs is technically challenging, especially in primary cells and 3-dimensional (3D) organotypic cultures. We optimized the previously reported RILES miRNA-ON sensor system to visualize the spatial expression of miR-203 and miR-30a by fluorescence imaging in 2-dimensional and 3D cultures of human primary keratinocytes. The generated system, called RIFES (RNAi-inducible fluorescence expression system), successfully imaged the expression of miR-30a-5p and miR-30a-3p in the suprabasal layers of the epidermis. This information was exploited to uncover the molecular mechanisms regulating the expression of miR-30a in human keratinocytes. We demonstrate that chemical inhibition of the Notch1 pathway induced GFP expression in undifferentiated RIFES/miR-30a keratinocyte cells, with fluorescence redistribution in the basal layers of 3D RIFES/miR-30a epidermis. Moreover, overexpressing miR-30a in 3D epidermal models resulted in NOTCH1 downregulation, suggesting a negative feedback loop between miR-30a and Notch. Because the Notch pathway was found downregulated in aged epidermis biopsies, we propose that Notch downregulation contributes to miR-30a induction during aging. Therefore, the RIFES system appears as a powerful tool to visualize the expression of microRNAs in 3D epidermis and to identify their potential upstream regulators.

## Introduction

MicroRNAs (miRNAs) are small noncoding RNAs that post-transcriptionally regulate gene expression. They are considered as fine tuners of genes networks, contributing to their robustness and accuracy (Stark et al, 2005). miRNAs are involved in numerous physiological cellular processes such as proliferation, differentiation, intracellular signaling, DNA repair, or cell motility. In fact, it is considered that 60% of the whole transcriptome of human cells is controlled by miRNAs. Therefore, it is not surprising that miRNAs play vital roles in the homeostasis of the skin because specific genetic ablation in skin of 2 key components of miRNA biogenesis, *Dicer* or *Dgcr8*, are lethal for the transgenic mice owing to dehydration after epidermal barrier loss (Yi et al, 2009). In fact, it is now widely acknowledged that miRNA expression is profoundly altered in various cutaneous pathological conditions, including skin cancer, skin inflammatory diseases, or skin aging, and that they represent valuable biomarkers for diagnostic or response to treatments (Gerasymchuk et al, 2020; Liu et al, 2017; Mirzaei et al, 2016). Extensive studies have been carried out to identify miRNAs gene targets using bioinformatical tools (Madhumita and Paul, 2022; Rajewsky and Socci, 2004), OMICS methods (Sindhu et al, 2021), or dedicated experimental approaches (Ørom and Lund, 2010). However and surprisingly, the upstream regulatory mechanisms responsible for the modulation of miRNAs in specific pathological conditions remain relatively unexplored. This could be explained by several reasons. The first arises from the small size of miRNA molecules, which significantly hampers the development of accurate probes for precise quantification in tissues or cells. The second reason is because miRNA have a dynamic spatial and temporal expression pattern that are difficult to capture using conventionnal approaches, such as reverse transcription quantitative PCR (RT-qPCR), for instance. Furthermore, not all miRNA molecules present in cells are functional, meaning that they are not all engaged in their negative post-transcriptional regulator function (Chan and Slack, 2006; Mullokandov et al, 2012). As a result, detecting a pool of miRNA in cells does not guarantee that all miRNAs are biologically active, which leads to incorrect interpretations.

To address these issues, a miRNA-ON monitoring system called RILES (for RNAi-inducible luciferase expression system) was previously developed (Ezzine et al, 2013). This system relies on the engineering of an inducible expression system to switch ON the expression of the Firefly luciferase reporter gene when a miRNA of interest is expressed. The RILES system provides a temporal and spatial resolution expression pattern of miRNA in cells and in animal models of pathologies (Baril and Pichon, 2016; Simion et al, 2017) that conventional detection methods were unable to achieve. In addition, the RILES system monitors specifically the pool of miRNA functionally engaged in the RISC machinery rather than the bulk of miRNA detected within cells as demonstrated recently by AGO2-RIP assay (Henriet et al, 2023). However, the performance of the RILES system in primary culture of human cells, which are known to be difficult to transfect, has not been evaluated as well as its implementation in 3-dimensional (3D) organotypic cultures to address the spatial and temporal expression pattern of miRNAs.

In this work, we subcloned the GFP reporter gene in the RILES to generate a RIFES system, standing for RNAi-Inducible Fluorescence Expression System. We then evaluated the RIFES system in 3D skin tissues in a context of skin aging. Skin aging is a complex multifactorial mechanism leading to various cellular dysfunctions, such as profound modifications of epigenetic regulation networks (López-Otín et al, 2013). Expression changes of miRNA are frequently observed in aged tissues, including the skin (Gerasymchuk et al, 2020). A study by Muther et al (2017) showed that miR-30a-3p and -5p are upregulated in the epidermis of aged patients as well as in cultured keratinocytes isolated from aged skin (Muther et al, 2017). miR-30a ectopic expression in reconstructed epidermis impacts keratinocytes differentiation and mitophagy, increases apoptosis, and finally leads to a strong defect in epidermal barrier function, similar to what is observed in real aged skin (Chevalier et al, 2022; Muther et al, 2017). However, the mechanism governing the upregulation of miR-30a expression in aged human skin remains unknown. In this work, we aimed to clarify this point. To do so, we first evaluated the spatiotemporal expression of miR-30a-3p/5p in fully stratified reconstructed epidermis using the RIFES system. We took advantage of this powerful system to identify miR-30a upstream regulators and further studied their activity in human skin samples of aged donors. Thus, we demonstrated in this study that monitoring the spatiotemporal expression pattern of miRNA in 3D skin tissues is both feasible and valuable for investigating the mechanisms regulating their spatial and temporal expression. This opens up the prospect of applications in many other contexts, enabling significant progress to be made in understanding the mechanisms through which miRNAs are regulated in the skin.

## Results

### Validation of a stable fluorescent reporter gene version of the RILES system for miR-30a activity in primary keratinocytes

**Figure 1:**
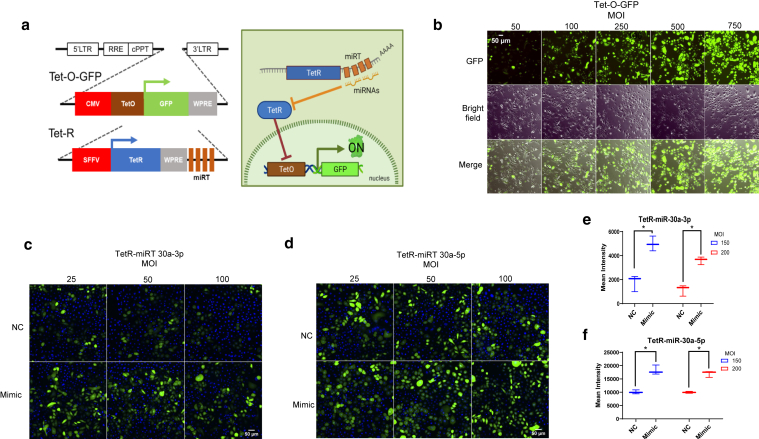
**Validation of stable RIFES reporter system for miR-30a activity.** (**a**) Distribution of the lentiviral RIFES elements in 2 vectors and schematic view of the activation of the GFP reporter gene in the presence of the microRNA of interest. (**b**) Images of the transduction of the Tet-O-GFP vector in HPK using different MOI. (**c, d**) Images of HPKs after a second transduction with the Tet-R-miRT vectors for (**c**) miR-30a-3p and (**d**) 5p at different MOI (25, 50, or 100) in normal control condition (NC) or after transfection with the corresponding miR-30a mimic (Mimic). Nuclei are stained in blue by DAPI. (**e**) Quantification of the fluorescence by HCS of the doubly transduced HPKs (with a MOI of 150 or 200 for the Tet-R-miR-30a-3p lentiviral vector) after transfection by the miR-30a-3p mimic (box plot with Tukey whiskers, n = 3; ∗*P* < .05 *t*-test *P*-value). (**f**) Quantification of the fluorescence by HCS of the doubly transduced HPKs (with a MOI of 150 or 200 for the Tet-R- miR-30a-5p lentiviral vector) after transfection by the miR-30a-5p mimic (box plot with Tukey whiskers, n = 3, ∗*P* < .05 *t*-test *P*-value). HCS, high-content screening; HPK, human primary keratinocyte; MOI, multiplicity of infection.

Because the main goal of our study was to monitor the expression of miRNAs in a reconstructed epidermis culture for several days, we needed a stable expression vector rather than a transitory expression plasmid. Moreover, because we wanted to determine the spatial localization pattern of 2 distincts miRNAs within the several suprabasal layers of epidermis, we replaced the Firefly Luciferase reporter gene of the original RILES system by the GFP reporter gene. This version of the RILES system was called RIFES for RNAi-inducible fluorescence system. These RIFES elements were thereafter subcloned into 2 lentiviral vectors originally used for the RILES system (Simion et al, 2021). The generated RIFES elements were divided into the 2 lentiRIFES vectors as follows: one contained *GFP* under regulation of the Tet-Operator (Tet-O-GFP vector), and the other had the repressor under the miRNA regulation (Tet-R-miRT) (Figure 1a). In total, 3 Tet-R-miRT lentiviral vectors containing each 4 complementary block sequences to bind to either miR-203-3p, miR-30a-3p, or miR-30a-5p were generated. As control, we used a Tet-R lentiviral vector without any miRNA-targeting sequence. Human primary keratinocytes (HPKs) were transduced with increasing multiplicity of infection (MOI) of Tet-O-GFP (Figure 1b). Next, after 1 cell passaging, those Tet-O-GFP–transduced HPKs were transduced again with the Tet-R-miR30a-3pT or -5pT vectors, resulting in an inhibition of the reporter gene expression because of endogenous expression of the Tet-R repressor protein in transduced cells (Figure 1c and d, top images). As expected, the fluorescence of the transduced cells was reactivated by miR-30a mimics transfections (Figure 1c and d, bottom images). Because the GFP inhibition was not complete, we transduced the HPK cells with a constant MOI value of 500 for the Tet-O-GFP lentivirus vector and with 2 different MOIs values (eg, 150 and 200) for the Tet-R-miR-30a-3p and Tet-R-miR-30a-5p lentivirus vectors. After transduction, we quantified the fluorescence signal emitted from living RIFES HPKs using a high-content screening assay. miRNA mimics were used as positive control to induce the expression of GFP. As shown in Figure 1e, the fluorescence intensity was significantly higher in mimics-transfected HPK both in lentiRIFES/miR-30a-3pT and in lentiRIFES/miR-30a-5pT cells, demonstrating the induction of the GFP reporter gene by RNA interference. We decided to work with 150 and 200 MOI for miR-30a-3p and miR-30-5p Tet-R-miRT vectors, respectively, and 500 MOI for Tet-O-GFP because the signal-to-noise ratio was optimal under those conditions.

### Development of a 1-step transduction of the lentiRIFES system in HPKs

**Figure 2:**
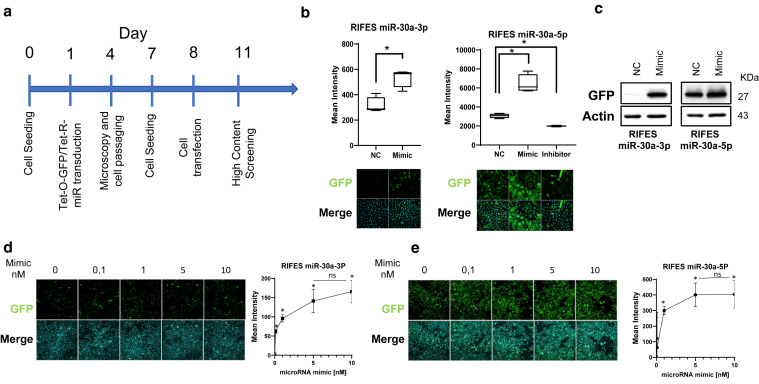
**Validation of lentiRIFES miR-30a system by a 1-step transduction of the 2 lentiviral vectors.** (**a**) Timeline for the cotransduction of the TetO-GFP and Tet-miRT vectors in a single step at day 1 followed by a transfection of the microRNA mimic (10 nM) or microRNA inhibitor at day 8. (**b**) The fluorescence was quantified by HCS at day 11. NC denotes normal control condition (box plot with Tukey whiskers, n = 4, ∗*P* < .05 *t*-test *P*-value). Representative images of the corresponding HPK cultures are shown under the plot (merge image: GFP plus DAPI). (**c**) Western blot analysis of GFP and actin expression in RIFES miR-30a HPKs after transfection by miR-30a-3p or 5p mimic. (**d**) The sensitivity of the lentiRIFES/miR-30a-3pT was determined by transfection of increasing doses of miRNA-30a- 3p mimics in lentiRIFES/miR-30a-3pT HPKs. Representative images of the cells are shown on the left; plot on the right corresponds to GFP fluorescence quantification by HCS (mean ± SD, n = 3, ∗*P* < .05). One-way ANOVA with Tukey posthoc tests was used for the comparison with the 0 nM condition. ns denotes nonsignificant difference between the 5 nM and the 10 nM condition. (**e**) The sensitivity of the lentiRIFES/miR-30a-5pT was determined by transfection of increasing doses of miR-30a-5p mimics in lentiRIFES/miR-30a-5pT HPKs. Representative images of the cells are shown on the left; plot on the right corresponds to GFP fluorescence quantification by HCS (mean ± SD, n = 3, ∗*P* < .05). One-way ANOVA with Tukey posthoc tests was used for comparison with the 0 nM condition. ns denotes nonsignificant difference between the 5 nM and the 10 nM condition. HCS, high-content screening; HPK, human primary keratinocyte; miRNA, microRNA.

We next attempted to simplify these steps by performing a 1-step double transduction with the Tet-O-GFP and TetR-miRT lentivirus vectors using the optimal established MOI (Figure 2a). This 1-step approach is a key advantage in limiting the passage of primary culture cells and, therefore, to increase the chance of monitoring the miRNA in 3D specimen. We validated this protocol by analyzing the effect of miRNA mimics transfection in double LentiRIFES-transduced HPK cells. As previously reported, the induction of the GFP reporter gene from the lentiRIFES was observed when cells were transfected with the specific miR-30a mimic (Figure 2b). Conversely, transfection of a miR-30a-5p synthetic inhibitor reduced the expression of GFP in LentiRIFES/miR-30a-5pT HPK cells (Figure 2b). The same results were observed when the experiments were conducted using immunoblotting: higher GFP protein levels were detected in the 2 LentiRIFES cells after transfection of miRNA mimics (Figure 2c). To assess the sensitivity of the generated RIFES system, LentiRIFES cells were transfected with increasing doses of miRNA mimic. The results revealed a clear correlation between fluorescence intensity and the concentration of transfected miR-30a-3p mimic in the 0–5 nM range (R^2^ = 0.856) (Figure 2d). The dose–response curve generated after transfection of miR-30a-5p mimic showed also an acceptable correlation (R^2^ = 0.85) (Figure 2e). At higher concentrations, precisely at 10 nM, no statistically significant change in GFP expression was detected for both transfected miR-30a mimics. This demonstrate that the RIFLES system reaches saturation at a concentration equal or superior at 5 nM of transfected miRNA mimic. Furthermore, the LentiRIFES/miR-30a-3pT cells exhibited a higher sensitivity than LentiRIFES/miR-30a-5pT cells. In fact, transfection of 10 nM of miR-30a mimic increased by 2-fold the fluorescence intensity in LentiRIFES/miR-30a-3pT cells compared with 1-fold in same condition but in LentiRIFES/miR-30a-5pT cells (Figure 2b and c). Such difference could be explained by the differential basal expression level of miR-30a-5p and miR-30a-3p strands in cells (Muther et al, 2017), which induce a differential fluorescence expression level in LentiRIFES/miR-30aT cells. This was confirmed by the measure of the GFP intensity of the 2 lentiRIFES reporter constructions in basal control conditions, observed by image analysis (Figure 2b, normal control condition) or by GFP immunoblotting (Figure 2c, normal control condition).

### Monitoring miR-203 expression in HPKs and reconstructed epidermis

**Figure 3:**
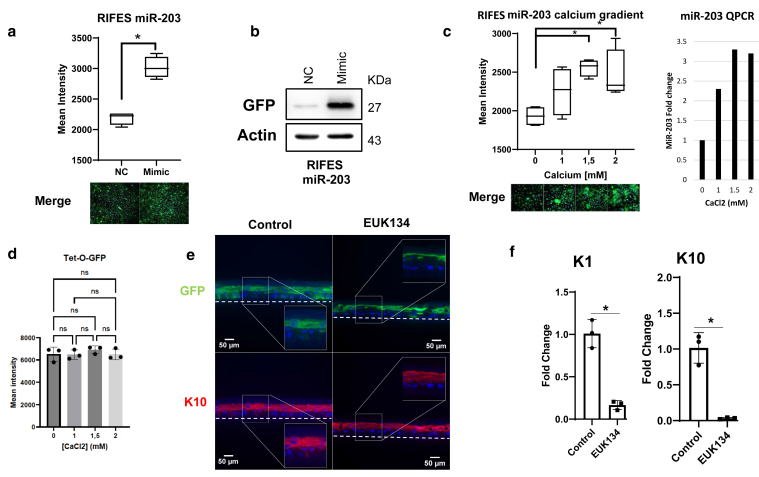
**Monitoring miR-203-3p activity in 2D and 3D HPK cultures.(a)** Validation of the lentiRIFES/miR-203-3pT after transfection of doubly transduced HPKs by miR-203-3p or scramble mimic (denoted as NC). The graph corresponds to the quantification of the GFP fluorescence by HCS (box plot with Tukey whiskers, n = 4, ∗*P* < .05 *t*-test *P*-value). A representative image of cells is shown (Merge image: GFP plus DAPI). (**b**) Western blot analysis of GFP and actin expression in lentiRIFES/miR-203T HPKs after transfection by miR-203-3p or scramble mimic (denoted as NC). (**c**) Response of the lentiRIFES/miR-203T HPKs to calcium treatment. Representative images of cells are shown (Merge image: GFP plus DAPI). The left graph corresponds to the quantification of the GFP fluorescence by HCS at various doses of calcium (box plot with Tukey whiskers, n = 4, ∗*P* < .05). One-way ANOVA with Tukey posthoc tests was performed. The right graph corresponds to the miR-203 relative expression addressed by qPCR after calcium treatment in HPKs (1 representative experiment is shown). (**d**) Effect of calcium treatment on the GFP fluorescence in HPKs transduced with the Tet-O-GFP lentivector (mean ± SD, n = 3). (**e**) Reconstructed epidermises were obtained using RIFES miR-203 keratinocytes. They were treated or not by the EUK134 compound. Representative images of GFP (**f**) K1 fluorescence or K10 immunofluorescence were shown. The limit of the epidermis is indicated by a dotted line. Bar = 50 mm. The frame shows a zoom on a part of the image and K10 transcripts relative expression analysis by qRT-PCR in HPKs treated or not by EUK134 (mean ± SD, n = 3, ∗*P* < .05 *t*-test *P*-value). 2D, 2-dimensional ; 3D, 3-dimensional; HCS, high-content screening; HPK, human primary keratinocyte; K, keratin; ns, nonsignificant.

Having demonstrated the performance of our LentiRIFES system in primary cultured cells, we next evaluated its feasibility to monitor the spatial expression pattern of miRNAs in 3D epidermal tissues. At first, we produced a LentiRIFES vector for monitoring the expression of miR-203-3p (LentiRIFES/miR-203T), a miRNA known to be strongly induced in differentiated human keratinocytes (Lena et al, 2008). As shown earlier, we validated the performance of the LentiRIFES/miR-203T system by transfection of a miR-203-3p mimic or a scrambled negative control miRNA sequence. As shown in Figure 3a, the generated stable HPK LentiRIFES/miR-203T cells transfected with miR-203-3p mimic showed a significant increase of the GFP fluorescence intensity that correlated well with a strong increase in GFP protein level detected by immunoblot (Figure 3b). Then, we evaluated whether GFP fluorescence could be induced in LentiRIFES/miR-203T cells induced to differentiate by calcium shift. As expected, the HPK LentiRIFES/miR-203T cells exhibited a higher fluorescence intensity after calcium-induced differentiation at concentrations above 1.5 mM (Figure 3c, left panel), in line with the miR-203 expression pattern addressed by qPCR (Figure 3c, right panel). To completely validate those results, we checked that the Tet-O-GFP itself was not responding to calcium treatment: we did not detect any significant variation of the GFP intensity in HPKs transduced with Tet-O-GFP and treated for 48 hours with different concentrations of calcium (Figure 3d). Once the lentiRIFES for miR-203 was validated in monolayer cultures, we next attempted to resolve the spatial expression of miR-203-3p within the layers of the 3D epidermis. We used a fibroblast-free 3D epidermal model obtained after 12 days of culture at the air–liquid interface and showing a full stratified epidermis (Figure 3e). Remarkably, GFP reporter activity was observed in suprabasal layers of the reconstructed human epidermis (RHE), similar to the differentiation marker keratin 10, in accordance with previous reports (Lena et al, 2008; Yi et al, 2008), showing that this miRNA is spatially expressed in the differentiated keratinocyte cells (Figure 3e). In addition, we tested the effect of EUK134, an antioxidant agent also known as a potent inhibitor of the epidermal differentiation (Monteleon et al, 2018). In the RHE treated with EUK134, a reduction of miR-203-3p GFP reporter signal and differentiation markers keratin 1 and keratin 10 was observed (Figure 3e and f). These results indicated that the LentiRIFES system is robust and specific to monitor the activity of a miRNA by fluorescence imaging in primary epidermal cultures as well as 3D reconstructed epidermis.

### miR-30a is expressed in suprabasal layers of 3D reconstructed epidermis, and its expression is reduced by NOTCH1 inhibition

**Figure 4:**
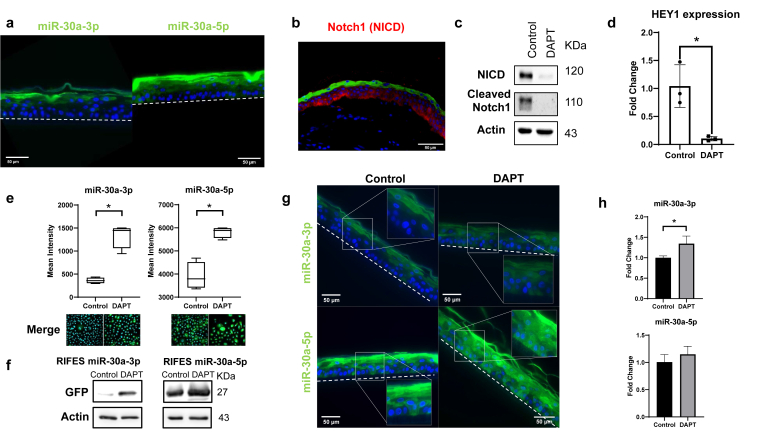
**The inhibition of the Notch pathway increases miR-30a activity.(a**) Representative images of reconstructed epidermises obtained using RIFES miR-30a-3p or miR-30a-5p primary keratinocytes. The green fluorescence corresponds to GFP, and the blue fluorescence corresponds to nuclei labeled with DAPI. The limit of the epidermis is indicated by a dotted line. Bar = 50 mm. (**b**) Immunofluorescence labeling of Notch 1 (denoted as NICD) in a skin biopsy (female abdominal skin, young adult). The red fluorescence corresponds to Notch1, the green fluorescence corresponds to loricrin, and the blue fluorescence corresponds to nuclei labeled with DAPI. Bar = 50 mm. (**c**) Western blot analysis of Notch1 (denoted as NICD), cleaved Notch1, and actin in HPKs treated or not by DAPT. (**d**) *HEY1* transcript relative expression analysis by qRT-PCR in HPKs treated or not by DAPT (mean ± SD, n = 3, ∗*P* < .05 *t*-test *P*-value). (**e**) Response of the RIFES miR-30a-3p or -5p HPKs to DAPT treatment. The graph corresponds to the quantification of the GFP fluorescence by HCS after DAPT or control treatment (box plot with Tukey whiskers, n = 4, ∗*P* < .05 *t*-test *P*-value). Representative images of cells are shown (Merge image: GFP plus DAPI). (**f**) Western blot analysis of GFP and actin expression in lentiRIFES/miR-30a-3pT or 5pT HPKs after DAPT treatment. (**g**) Reconstructed epidermises were obtained using lentiRIFES/miR-30a-3pT or 5pT keratinocytes. The RHEs were treated or not by the DAPT compound. Representative images of GFP fluorescence or K10 immunofluorescence are shown. The limit of the epidermis is indicated by a dotted line. Bar = 50 mm. A small area of the images surrounded by a frame is shown at higher magnification. (**h**) miR-30a-3p or miR-30a-5p transcript expression analysis by qRT-PCR in HPKs treated or not by DAPT (mean ± SD, n = 3, ∗*P* < .05 *t*-test *P*-value). HCS, high-content screening; HPK, human primary keratinocyte; K10, keratin 10; RHE, reconstructed human epidermis.

Next, we pursued our investigation by attempting to monitor the expression of miR-30a-3p and miR-30a-5p in 3D cultures by fluorescence imaging. Following the same methods described for miR-203-3p, we observed that GFP signals emitted from the LentiRIFES/miR-30a-3pT and LentiRIFES/miR-30a-5pT were detected in most suprabasal layers of the epidermal cultures (Figure 4a). Interestingly, similar to our observations in 2-dimensional culture (Figure 2), GFP signals emitted from RHE produced with the LentiRIFES/miR-30a-5pT cells were higher than those from RHE produced with the LentiRIFES/miR-30a-3pT system, confirming the higher expression of the 5p strand in human keratinocytes.

Next, we took advantage of this spatial expression pattern of miRNA-30a-3p/5p in the most suprabasal layer of the epidermis to investigate the molecular mechanism responsible for expression of this miRNA during the late differentiation program of keratinocyte cells. It has been previously reported that miR-30a expression is repressed by the Notch pathway in B- and T-cell malignancies (Ortega et al, 2015). Furthermore, in epidermis, the NOTCH1 receptor expression decreases in the most differentiated layers (Figure 4b), contrary to miR-30a-3p/5p expression. These 2 observations tend to suggest that miR-30a might be under negative regulation of Notch signaling in human keratinocytes. To explore this hypothesis, Notch pathway was inhibited in HPK cells after treatment with the small-molecule DAPT (N-[N-(3, 5-Distill-3, 5-dimethylbenzoyl)-L-alanyl]-S-phenylglycine tert-butyl ester). As expected, DAPT treatment decreased the protein levels of the NOTCH1 receptor and of the cleaved activated domain of NOTCH1 as well as downregulation of the HEY1 transcriptional factor, a downstream target of NOTCH1 (Figure 4c and d). Interestingly, the fluorescence expression patterns of miR-30a-3p and miR-30a-5p detected in HPK LentiRIFES/miR-30a-3pT and -30a-5pT cells were both induced after treatment with DAPT (Figure 4e and f). These support our hypothesis that Notch signaling negatively modulates the miR-30a-3p/5p expression. Of note, DAPT treatment of HPK cells transduced with the LentiRIFES control (LentiRIFES/miRCTL) did not induce any change in fluorescence induction (data not shown). Then, we analyzed the expression of miR-30a-3p/5p in 3D epidermal cultures produced with the HPK LentiRIFES/miR-30a-3pT and LentiRIFES/miR-30a-5pT cells and after Notch inhibition by DAPT. Strikingly, the spatial expression pattern of GFP drastically changed under this condition. GFP-emitted signals from these RIFES-engineered RHEs were now detected in the basal layers of 3D tissues compared with that of the control (Figure 4g). To validate the accuracy of this finding, we analyzed the expression of miR-30a-5p and -3p by RT-qPCR in the 2-dimensional keratinocyte cultures. Significantly increased levels of miR-30-3p were detected after treatment with DAPT, whereas no change in expression of miR-30a-5p was found (Figure 4h). All together, these data indicate that the expression of miR-30a is under the negative control of the Notch pathway in keratinocyte cell cultured in 2 dimensions and 3D.

### Validation of the regulatory loop NOTCH–MYC–miR-30a

**Figure 5:**
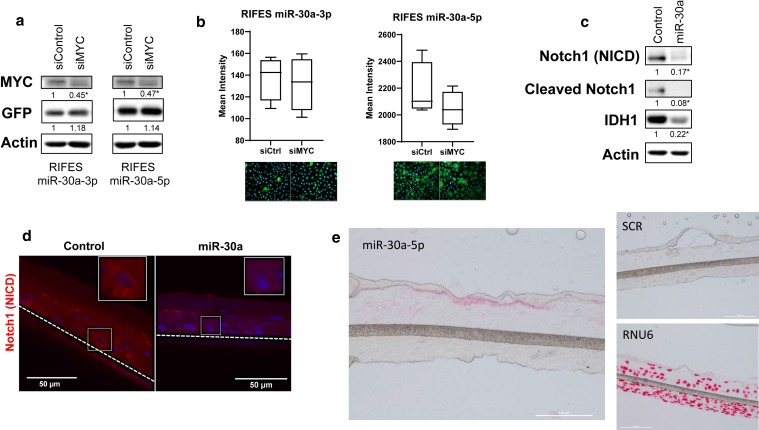
**Validation of a negative feedback loop between miR-30a and Notch.(a**) Western blot analysis of Myc, GFP, and b-actin expression in lentiRIFES/miR-30a-3pT or 5pT keratinocytes transfected with an siRNA targeting Myc. The relative protein expression of siMYC versus siCtrl normalized to b-actin is indicated below the blot image (∗*P* < .05 *t*-test *P*-value). (**b**) Response of the lentiRIFES/miR-30a-3pT or -5pT HPK to siMYC. The graph corresponds to the quantification of the GFP fluorescence by HCS after siMYC or siRNA control treatment (box plot with Tukey whiskers, n = 4, ∗*P* < .05 *t*-test *P*-value). Representative images of cells are shown (merge image: GFP plus DAPI). (**c**) Western blot analysis of NOTCH1 (NICD), cleaved NOTCH1, IDH1, and b-actin expression in keratinocytes transfected with a lentivirus leading to stable miR-30a overexpression. The relative protein expression of miR-30a versus control normalized to b-actin is indicated below the blot image (∗*P* < .05 *t*-test *P*-value). (**d**) NOTCH1 (NICD) immunofluorescence (red labeling) in reconstructed epidermis obtained with HPKs overexpressing miR-30a or control reconstructed epidermis. The limit of the epidermis is indicated with a dotted line. The nuclei are labeled in blue with DAPI. A small area of the image surrounded by a frame is shown at higher magnification. Bar = 50 mm. (**e**) miR-30a localization by in situ hybridization in RHE. RHEs were produced with human primary keratinocytes ectopically overexpressing miR-30a. In situ hybridization was performed with a miR-30a-5p probe, a RNU6 probe as positive control, or a nontargeting probe (denoted as SCR) as negative control. Bar = 100 mm. HCS, high-content screening; HPK, human primary keratinocyte; RHE, reconstructed human epidermis; siCtrl control-targeted small interfering RNA; siMYC, MYC-targeted small interfering RNA; siRNA, small interfering RNA.

Previous published results indicated that regulation of miR-30a by Notch pathway is mediated by c-MYC in B- and T-cell malignancies (Ortega et al, 2015). In addition, in these malignant cells, miR-30a was also found to negatively regulate NOTCH protein, resulting in an autoregulatory loop to maintain accurate expression level of Notch in these cells (Ortega et al, 2015). To investigate whether this regulatory axis might also occur in keratinocytes, c-MYC expression was silenced in the HPK LentiRIFES/miR30a-3pT or -5pT stable cell lines. As shown in Figure 5a, small interfering RNA silencing of c-MYC did not impact on miR-30a expression because GFP-emitted signals as well as GFP protein level were unchanged in those cells (Figure 5a and b, respectively), indicating that miR-30a is not regulated by c-MYC in keratinocytes.

To further support the negative regulatory interplay between miR-30a and NOTCH expression, HPKs were transduced by a lentivirus carrying the miR-30a gene, leading to miR-30a-3p and miR-30a-5p overexpression (data not shown). As control, the HPK cells were transduced with a lentivirus encoding for a scrambled control miRNA. Because NOTCH1 is a protein involved in differentiation, HPK differentiation was induced by calcium. As shown in Figure 5c, keratinocytes overexpressing miR-30a showed lower levels of Notch1 protein expression than the scrambled miRNA control cells. IDH1 was used as control because it had been previously validated as miR-30a target (Muther et al, 2017). As expected, IDH1 was downregulated in miR-30a–overexpressing HPK cells (Figure 5c). Finally, we produced a reconstructed epidermis with keratinocytes overexpressing miR-30a. In these miR-30a–overexpressed RHE, miR-30a-5p was visible by in situ hybridization (ISH) and revealed an expression pattern quite similar to that observed with the RIFES reporter system (Figure 4a), with a stronger labeling in the upper layers of the epidermis (Figure 5e). In the miR-30a–overexpressing RHE, a lower NOTCH1 expression was detected (Figure 5d), similar to that of the monolayer culture of differentiated HKP cells. All together, these results demonstrate that NOTCH1 is negatively regulated by miR-30a, and therefore, miR-30a and NOTCH form a regulatory negative feedback loop in human keratinocytes.

### NOTCH expression decreases in skin aging

**Figure 6:**
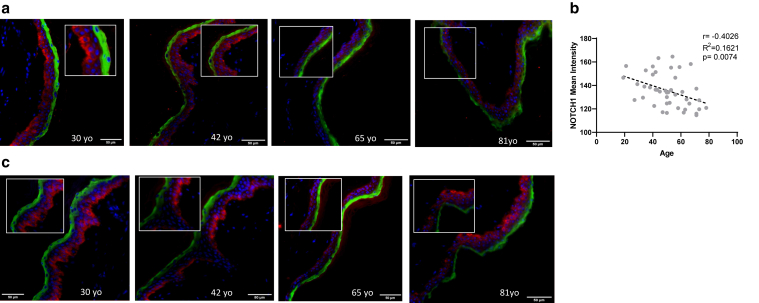
**Notch decreases with epidermal chronological aging.** (**a**) Immunofluorescence labelling of NOTCH1 (NICD) protein (red signal) in human skin tissue microarray. Representative images of 4 samples of different age are shown (a small area of the image surrounded by a frame is shown at higher magnification). The differentiation marker loricrin is labeled in green. Nuclei are stained in blue in DAPI. Bar = 50 mm. (**b**) Inverse correlation between NOTCH1 (NICD) expression level (mean intensity measured by ImageJ) and skin donor age for the samples of the tissue microarray. (**c**) Immunofluorescence labeling of cleaved NOTCH1 (red signal) in human skin samples of different ages (a small area of the image surrounded by a frame is shown at higher magnification). The differentiation marker loricrin is labeled in green. Nuclei are stained in blue by DAPI. Bar = 50 mm.

Because it was previously reported that miR-30a is an aging-related miRNA (Muther et al, 2017), we therefore analyzed NOTCH1 expression by immunofluorescence in 40 skin samples from donors of different ages and observed reduced NOTCH1 expression in aged samples (Figure 6a). As shown in Figure 6b, total NOTCH1 expression showed a negative correlation with respect to age. We also analyzed the expression of cleaved NOTCH1 in skin samples for individuals of different ages and observed a tendency to be reduced in term of distribution and intensity (Figure 6c). These data demonstrate that NOTCH1 expression decreases in epidermal aging. Therefore, according to our study, this downregulation of the Notch pathway could contribute to miR-30a overexpression in aged epidermis.

## Discussion

Conventional miRNA-profiling techniques, including Northern blot, real-time PCR, microarray, ISH, and deep sequencing, have significantly advanced our understanding of miRNA biology. However, these methods rarely provide insights into the real-time spatiotemporal organization and regulatory activity of miRNAs mainly because of their destructive nature, which impairs the visualization of miRNA localization within tissues or organs and especially during longitudinal studies. The RILES system, a luciferase reporter system based on RNA interference driven by a miRNA of interest, has been previously designed to overcome such hurdles and to gain information on the temporal and functional regulatory activity aspect of miRNA in hurdlers vivo and in cellulo models of pathologies without the need for sample lysis (Ezzine et al, 2013; Simion et al, 2020, 2017). Furthermore, the RILES system was recently successfully employed as a miRNA-cell–based screening platform to identify modulators of epidermal homeostasis with a possible therapeutic application in psoriasis management (Henriet et al, 2023).

In this article, we used a modified version of the RILES system delivered by lentiviral virus to transduce HPKs with the aim to generate long-term stable expression of a fluorescent version of RILES, called RIFES. We placed the RIFES system under the control of miR-203, miR-30a-3p, or miR-30a-5p selected for their important role in keratinocyte differentiation (He et al, 2019; Lena et al, 2008). We demonstrated that it is possible to generate a 3D epidermal culture expressing the RIFES system for several days without compromising the histology of epidermal layers. We successfully monitored the spatial expression of 3 different miRNAs in epidermal layers of 3D RHE. We confirmed that miR-203 regulatory activity was restricted to the upper part of the human epidermis, in concordance with its localization previously addressed by ISH (Wei et al, 2010). We also monitored the spatial expression of miR-30a-3p and miR-30a-5p. We demonstrated that these 2 miRNAs were also mostly localized in the suprabasal layer of the epidermis.

However, although the RIFES system has proven its efficiency for monitoring the spatial expression patterns of miRNAs in 3D tissues, this GFP-based miR-ON reporter system has some limitations. The first, which was also observed with the RILES version employing the Firefly luciferase reporter (Ezzine et al, 2013; Simion et al, 2017), is that the dynamic range of reporter induction in response to increasing concentrations of transfected miRNA is linear at low concentrations, typically from 1 to 5 nM. Beyond this concentration, the RIFES system reaches saturation, possibly owing to an overload of the RISC machinery that thereby limits its ability to repress the abundant amount of TetR mRNA present in cells. The second limitation of the RIFES system is that, similar to any other protein, the eGFP reporter has an intrinsic half-live. Although we used an enhanced GFP variant with an approximate half-life of 8 hours, compared with 24 hours for the original GFP, the RIFES system is not expected to effectively monitor change in miRNA expression occurring over shorter time frame owing to the persistence of the eGFP protein in cells for at least less than 8 hours. Owing to these limitations, it can be assumed that the RIFES system, as we have used it in this study, cannot fully establish the absolute quantity of miRNAs involved and is better considered as qualitative miRNA monitoring system rather than quantitative. We are currently developing of complete program of work to address these drawbacks.

However, it is worth noting that despite of these limitations, the use of a GFP-based miRNA-ON reporter system offers some advantages compared with the conventional methods used to detect miRNA in tissue sections as miRNA ISH. We have demonstrated that the RIFES/30a probe is significantly more effective in detecting miR-30a-5p and -3p in tissue sections of reconstructed 3D epidermis than fluorochrome-labeled RNA probes designed for ISH. Indeed, it was possible to detect miR-30a by ISH in RHE but only when miR-30a was ectopically overexpressed. This enhanced efficiency of the RIFES compared with that of ISH may be attributed to the fact that the RIFES system is switched ON by the endogenous catalytic RISC complex in cells and that reporter genes amplify signals to a much greater extent than miRNA probes, which are short in size, limiting the number of fluorophores that can be chemically attached.

miR-30a has been previously described as a key regulator of chronological aging by modulating mitophagy during keratinocytes differentiation, impairing epidermal homeostasis in aged skin (Chevalier et al, 2022; Muther et al, 2017). However, the mechanism of miRNA-30a regulation during aging was not described. The 3D spatial expression patterns of miR-30a-3p and miR-30a-5p generated by the RIFES system were therefore exploited to gather insights into the regulatory mechanisms of expression of miR-30a. Attempting that miR-30a is mostly expressed in suprabasal layers of epidermis, we focus our investigation on known molecular modulators of terminal differentiation. NOTCH appeared as a good candidate because its role during the late differentiation process of keratinocytes was already known (Koh et al, 2015) and because miR-30a has been found to be regulated by NOTCH in other cell types (Ortega et al, 2015). In this study, we brought experimental evidence that expression of miR-30a is negatively regulated by the Notch pathway in primary keratinocyte and this, specifically within a context of aging process. This opposite regulation is further supported by the observation that, unlike miR-30a, the expression of the NOTCH receptor is repressed in aged epidermis. Notably, we established this negative correlation between NOTCH expression and aging using a large number of skin samples, aligning with previous reports (Palazzo et al, 2015). This suggest a decreased activity of the Notch pathway with aging, which could explain the observed overexpression of miR-30a in aged cultured keratinocytes and aged epidermis samples (Muther et al, 2017). Alteration of Notch signaling with aging has been observed in various organs, including muscle (Carey et al, 2007) and neurovascular system (Kapoor and Nation, 2021), inducing dysfunctions and age-related diseases.

The mechanisms of miR-30a regulation by NOTCH are further complicated to investigate by the fact that miR-30a and Notch establish a negative feedback loop. We indeed observed that overexpression of miR-30a decreases NOTCH1 expression, whereas inhibition of the Notch pathway increases miR-30a. Regulatory feedback loops involving miRNAs have been frequently described, especially in the skin for the control of skin development and differentiation. It is the case for miR-203 with targets *p63* (Candi et al, 2015), which in turn regulates DICER (Boominathan, 2010), a key activator of the miRNA synthesis pathway. It has been shown previously that the fine regulation of Notch signaling pathway can be ensured by similar mechanisms involving miRNAs in various cell type such as neural progenitors (Bonev et al, 2012; Roese-Koerner et al, 2017) or osteoblasts (Lina et al, 2019). This type of regulatory loop reinforces the stability and robustness of genetic networks (Stark et al, 2005), particularly for signaling pathways that play key roles in tissue homeostasis, such as the Notch pathway in the epidermis. What remains to be understood is how this regulatory loop is disrupted during chronological aging, leading to overexpression of miR-30a in aged epidermis.

In conclusion, in this work, we establish a protocol to engineer a powerful tool for use in 2-dimensional cultures of primary keratinocytes as well as in 3D models of reconstructed epidermis. This tool enables the monitoring of the spatial and temporal activity of a specific miRNA through GFP imaging in a 3D format without the need to lyse the sample. Beyond the data generated in this study, the RIFES system, when combined with cell sorting and single-cell sequencing approaches, should enable the characterization of molecular signatures of cells expressing a miRNA of interest at an unequalled single-cell level. Such approach is invaluable for gaining a deeper understanding of how a miRNA can influence the behavior and fate of a single cell within its tissue environment.

## Materials and Methods

### Plasmid constructions and lentiviral production

RILES plasmids for miR-30a-3p and miR-30-5p were donated by Patrick Baril’s laboratory. Furthermore, the miRT cassettes were subcloned into the lentiviral vector TetR (lead to TetR-miRT vector). The miRT cassettes contained 4 complementary reverse sequences to the mature miRNA strand of interest as binging sites and a Nhe I restriction site in the 3′ end. The miRT cassettes were amplified by PCR from the RILES plasmids for miR-30a-3p and miR-30a-5p, adding a Xho I site in each end, whereas for miR-203, 2 complementary oligonucleotides (Sigma-Aldrich) were annealed by heating for 5 minutes and cooling for 45 minutes at room temperature. All the miRT cassettes sequences and primers for their amplification are listed in Table 1. Later, miRT cassettes and TetR plasmid were digested with Xho I enzyme (Thermo Fisher Scientific), purified by gel, and quantified. In addition, the TetR vector was dephosphorylated with Quick CIP (New England Biolabs). Then, the miRT cassettes were ligated to the linearized plasmids with the Quick Ligase (New England Biolabs) and used for transforming competent bacteria (New England Biolabs). The constructions were verified by Nhe I restriction and sequencing. Later, they were used for the lentiviral particles production at the vector facility of the SFR Biosciences Gerland-Lyon Sud (Lyon) as previously described (Muther et al, 2017).

**Table 1:** Oligonucleotides for miRT Cassette Subcloning

Name	Sequence 5′-3′
miR-30a-3p forward subcloning	CTCGAGCTCGAGAAGCTGCAAACATCCG
miR-30a-3p reverse subcloning	CTCGAGCTCGAGAAGCTAGCCTTTCAGTCG
miR-30a-5p forward subcloning	CTCGAGCTCGAGAACTTCCAGTCGAGGATG
miR-30a-5p reverse subcloning	CTCGAGCTCGAGAAGCTAGCTGTAAACATCCT
Oligo miRT-203 cassette sense	TACCATGAGACTCGAGAACTAGTGGTCCTAAACATTTCACTAGTACTAGTGGTCCTAAACATTTCACCGATCTAGTGGTCCTAAACATTTCACATGCCTAGTGGTCCTAAACATTTCACGCTAGCTTCTCGAGTACCATGAGA
Oligo miRT-203 cassette antisense	TCTCATGGTACTCGAGAAGCTAGCGTGAAATGTTTAGGACCACTAGGCATGTGAAATGTTTAGGACCACTAGATCGGTGAAATGTTTAGGACCACTAGTACTAGTGAAATGTTTAGGACCACTAGTTCTCGAGTCTCATGGTA

### Monolayer culture

HPKs were isolated as previously described (Muther et al, 2017) from pediatric healthy donors with written informed consent of the parents according to the ethical guidelines (French Bioethics law of 2004) and declared to the French research ministry (declaration number DC-2008-162 delivered to the Cell and Tissue Bank of Hospices Civiles de Lyon). Briefly, foreskin samples were treated overnight with trypsin (Gibco, Thermo Fisher Scientific) and Dispase (Dispase II, Roche Diagnostics). The following day, epidermis was separated from the dermis using forceps. Then, trypsin was deactivated by addition of DMEM supplemented with 10% of fetal bovine serum and removed by centrifugation. Then, cells were cultured in KGM2 (Promocell), except for cells used for RHE, which were maintained in EpiLife medium (Thermo Fisher Scientific). All the cultures were routinely checked for mycoplasma contamination and maintained in a humified atmosphere at 5% carbon dioxide and 37 °C. For all the subsequent experiments, HPKs were used at early passage (2 or 3) at 70% of confluency.

### Transfections

Synthetic mirVana miRNA mimics (miR-30a-3p, miR-30a-5p, miR-203), an inhibitor (anti-miR miR-30a-5p), and a scrambled negative control were purchased from Ambion (Thermo Fisher Scientific). Control small interfering RNA and small interfering RNA for c-myc were purchased from Santa Cruz Biotechnology. RNAi transfection was performed at 20 nM or in a range of 0.1–10 nM for the sensitivity assays, using Invitrogen Lipofectamine RNAiMAX reagent (Thermo Fisher Scientific), following the manufacturer’s instructions. When RILES plasmids were cotransfected with the miRNA mimics, transIT-2X transfection reagent (Mirus Bio) was used at 1:2 TransIT-X2: DNA ratio and 10 nM miRNA mimics. In addition, a *Renilla* luciferase reporter plasmid was used as control for luminescence activity normalization (VectorBuilder, number VB191205-1069fzu). The effect of the transfection was evaluated 48 or 72 hours after transfection according to the assay.

### Luciferase assay

Luciferase activity was determined with the Dual-Glo Luciferase Assay System kit (Promega), according to the manufacturer’s directions. Briefly, 48 hours after transfection, HPKs were lysed for measuring the firefly signal first, and the *Renilla* signal was subsequently detected in a microplate reader (Infinite M1000, TECAN). Luciferase activity, represented as Relative Lights Units, was calculated as Firefly luciferase signal divided by *Renilla* luciferase signal.

### Lentiviral transduction of the RIFES vectors

The viral particles needed per MOI was calculated with the formula: (cell number seeded × MOI)/viral titer (IU/μl) = μl needed. Thus, HPKs were seeded in a 6-well plate and transduced overnight the following day, adding fresh medium the morning after transduction. Different MOIs doses were tested, having the best results at 600 MOI for the O-GFP vector, 150 MOI for the TetR-miRT miR-30a-3p, and 200 MOI for TetR-miRT miR-30a-5p and miR-203. The standardization is detailed in the results.

### DAPT treatment

The gamma-secretase inhibitor DAPT (Abcam) was used at final concentration of 50 μM for 72 hours in HPKs at 70% of confluency. For RHE, the treatment was performed at 50 μM for 24 hours at the day 9 of culture and 2 days more with fresh medium (Palazzo et al, 2015).

### RHE

RHEs were made as described previously (De Vuyst et al, 2014). Briefly, HPKs were cultured in EpiLife medium supplemented with HKGS (Cascade Biologics, Thermo Fisher Scientific). HPKs were harvested by trypsinization when cells reached 60–70% of confluency. Next, the pellet was resuspended in cold EpiLife containing 1.5 mM of calcium chloride at density of 300 cells/μl. Polycarbonate inserts for cell culture (0.4 μm pore size, area 0.63 cm^2^, Merck Millipore) were placed in 6-well plates filled with 2.5 ml EpiLife containing 1.5 mM calcium chloride, and then 500 μl of cell suspension were added in the upper chamber, corresponding to 250,000 cells. The following day, cells were exposed to the air–liquid interface and maintained in EpiLife containing 1.5 mM calcium chloride, 50 μg/ml vitamin C, and 10 ng/ml keratinocyte GF for 11 days more, changing medium every day. The stratified RHEs were recovered using a scalpel and embedded in paraffin. RHEs overexpressing miR-30a were produced as previously described (Muther et al, 2017).

### RT-qPCR assays

The relative quantification of mRNAs was made by 2-step RT-qPCR. In summary, total RNA was extracted using NucleoSpin RNA purification Kit (Macherey-Nagel). Retrotranscription was made using PrimeScript RT reagent kit (Takara Bio), and the quantification was performed with SYBR Premix ExTaqII (Takara Bio) in an AriaMx Realtime PCR system (Agilent Genomics). The TBP gene was used as reference control. The primers’ sequences are listed in Table 2. Relative expression quantification was estimated by 2^-ΔΔCT^ method. Primers for qPCR are listed in Table 2.

**Table 2:** RT-qPCR Primers

Name	Sequence 5′-3′
TBP forward	TCAAACCCAGAATTGTTCTCCTTAT
TBP reverse	CCTGAATCCCTTTAGAATAGGGTAGA
HEY1 forward	TGAATCCAGATGACCAGCTACTGT
HEY1 reverse	TACTTTCAGACTCCGATCGCTTAC
K1 forward	GATGAAATCAACAAGCGGACAA
K1 reverse	GGTAGAGTGCTGTAAGGAAATCAATT
K10 forward	TCCCCCTGATGTGAGTTGC
K10 reverse	GAATCTGAATGACCGCCTGG

RT-qPCR for miRNA quantification was made using TaqMan Probes for miR-30a-3p, miR-30a-5p, and miR-203-3p (Thermo Fisher Scientific), according to the manufacturer’s instructions. RNU 48 was used as a reference control.

### Western blot

Protein extracts from HPKs were prepared in RIPA buffer (50 mM Tris-hydrogen chloride, pH 8, 150 mM sodium chloride, 1.5 mM potassium chloride, 1% NP-40, 0.1% SDS, 0.5% sodium deoxycholate, 0.1% Triton X-100, 1 mM EDTA) supplemented with protease and phosphatase inhibitors (Roche). A total of 20 μg of protein were resolved on SDS-Polyacrylamide 4–8% gels and transferred onto polyvinylidene difluoride membranes (Merck Millipore). Membranes were blocked with 10% nonfat dry milk for 1 hour and incubated with primary antibodies against NOTCH 1 (number 3608, Cell Signaling Technology), cleaved NOTCH-1 (number 4147, Cell Signaling Technology), c-MYC, or β-actin (ab8227, Abcam) overnight at 4 °C. Membranes were incubated for 1 hour with anti-rabbit horseradish peroxidase–conjugated secondary antibodies at room temperature, for subsequently revealing with West Pico PLUS Chemiluminescent substrate (Thermo Fisher Scientific) in a Fusion Fx system (Vilber).

### High-content screening assays

HPKs were seeded and treated in black-transparent 96 well plates (Greiner Bio One). After treatment, HPKs were maintained in PBS with ProLong Live Antifade Reagent for live cell imaging (Thermo Fisher Scientific) and Hoechst staining (Thermo Fisher Scientific). Then, the plates were incubated in a Yokogawa CQ1 confocal system (Masashino) at 37 °C and 5% carbon dioxide. Then, the device was programmed for acquiring the green and the blue channels, 5 fields per well with the ×10 or ×40 magnification. After acquisition, using the CellPathFinder software, the mean intensity signal per cell was determined by segmentation of the nucleus (blue) and cytoplasm (green).

### Tissue immunofluorescence and ISH

Paraffin-embedded tissue microarray of human skin samples (US Biomax) and slides with paraffin-embedded RHE samples were rehydrated by immersion in xylene and ethanol. Later, the samples were permeabilized with Triton X-100 at 0.1% and blocked with PBS containing 5% goat serum, 2% BSA, and 0.1 % Tween 20 for 1 hour at room temperature. Then, slides were washed and incubated with primary antibodies (mentioned in the western blot details) overnight at 4 °C. The following day, slides were washed and incubated for 1 hour with Alexa-fluor secondary antibodies (Thermo Fisher Scientific) at room temperature. Finally, the sampled were prepared with Prolong Glass Antifade mounting medium with NucBlue (Thermo Fisher Scientific). For miR-30a staining by ISH in RHE paraffin sections, the miRNAscope HD (RED) Assay (Advanced Cell Diagnostics) was used with the SR-has-miR-30a-5p-S1 probe (Advanced Cell Diagnostics, ref 1006211-S1) following strictly the manufacturer’s instructions. Images were acquired with a Nikon TE300 microscope for RHE and with an automated Yokogawa CQ1 for the tissue microarrays. Post-acquisition analysis was done with CellProfiler (https://cellprofiler.org/) and ImageJ.

### Statistical analyses

Statistical analyses were performed on the GraphPad Prism 7.0 software (GraphPad). A *t*-test was used for comparison of 2 groups, and 1-way ANOVA with Tukey posthoc tests was used when comparing 3 or more experimental groups. In addition, Pearson correlation was estimated for the tissue microarray. Statistical significance was considered as *P* < .05 (indicated with ∗). Data are depicted as bar graphs of the mean with SD or by boxplot giving the median value and the limits of 1st and 3^rd^ quartile, with Tukey whiskers showing lower and upper adjacent values.

## Ethics Statement

Human primary keratinocytes were isolated from healthy pediatric donors with informed consent of the parents according to the ethical guidelines (French Bioethics law of 2004) and declared to the French research ministry (declaration number DC-2008-162 delivered to the Cell and Tissue Bank of Hospices Civils de Lyon).

## Data Availability Statement

The data that support the findings of this study are available from the corresponding author (Jerome.lamartine@univ-lyon1.fr) upon request.

## ORCIDs

Alejandro Gonzalez Torres: http://orcid.org/0000-0001-8270-4404

Fabien P. Chevalier: http://orcid.org/0000-0001-8034-8661

Ruth Aquino: http://orcid.org/0000-0001-7692-091X

Melanie Aimard: http://orcid.org/0009-0004-2990-4537

Patrick Baril: http://orcid.org/0000-0001-8290-9899

Jérôme Lamartine: http://orcid.org/0000-0003-2322-1618

## Conflict of Interest

The authors state no conflict of interest.
